# Inhibiting the P2X7R-NLRP3 inflammasome pathway regulates CXCL16 to alleviate podocyte injury in mice with adriamycin nephropathy

**DOI:** 10.1038/s41598-026-47345-5

**Published:** 2026-04-05

**Authors:** Yanji Zhu, Zihan Zong, Xiaoyi Li, Suwen Liu, Qian Li, Junhui Zhen, Shuzhen Sun

**Affiliations:** 1 Department of Pediatrics, Shandong Provincial Hospital Affiliated to Shandong First Medical University, Jinan, 250021 Shandong People’s Republic of China; 2 Medical Integration and Practice Center, Cheeloo College of Medicine, Shandong University, Jinan, 250012 Shandong People’s Republic of China; 3 Department of Pediatrics, People’s Hospital of Rizhao, Rizhao, 276800 People’s Republic of China; 4 Department of Pathology, Qilu Hospital of Shandong University, Cheeloo College of Medicine, Jinan, 250012 Shandong People’s Republic of China

**Keywords:** Primary nephrotic syndrome, Podocyte injury, P2X7R, NLRP3 inflammasome, CXCL16, Cell biology, Diseases, Nephrology

## Abstract

Primary nephrotic syndrome (PNS) is a leading cause of chronic kidney disease in children. Focal segmental glomerulosclerosis (FSGS) remains a clinical challenge in terms of treatment. To investigate the role of the P2X7R-NLRP3 inflammasome pathway in podocyte injury in PNS, focusing on its regulation of CXCL16 in FSGS. Clinical renal tissues from children with PNS were analyzed by immunohistochemistry for P2X7R, NLRP3, and CXCL16 expression. In vitro, adriamycin (ADR)-induced podocyte injury models were established, with P2X7R knockdown via lentiviral transduction. Protein and mRNA levels of pathway components and injury markers were assessed by Western Blot and RT-qPCR; IL-1β and IL-18 were measured by ELISA. In vivo, ADR nephropathy was induced with ADR 25 mg/kg by a single tail vein injection in NLRP3-knockout and wild-type male mice (n = 10 per group C57BL/6J, 5 weeks old). Serum albumin, total cholesterol, 24 h urine protein, renal expression of CXCL16, Nephrin, and inflammatory cytokines, and podocyte ultrastructure were evaluated. Glomerular P2X7R, NLRP3, and CXCL16 were significantly upregulated in PNS patients, particularly in FSGS versus MCD. ADR-induced podocytes showed increased expression of these markers and inflammatory cytokines, which P2X7R knockdown reversed. NLRP3 knockout in mice attenuated ADR nephropathy, improving biochemical parameters, reducing proteinuria, downregulating CXCL16 and inflammatory factors, restoring Nephrin, and ameliorating podocyte ultrastructural injury. The P2X7R-NLRP3 inflammasome pathway promotes podocyte injury in PNS via CXCL16 upregulation, revealing novel mechanistic insights and potential therapeutic targets.

**Supplementary Information:**

The online version contains supplementary material available at 10.1038/s41598-026-47345-5.

## Introduction

Primary nephrotic syndrome (PNS) represents one of the most prevalent glomerular disorders in children. The majority of pediatric PNS cases exhibit the pathological features of minimal change disease (MCD) and respond favorably to corticosteroid therapy, a condition termed steroid-sensitive nephrotic syndrome (SSNS), which is associated with a favorable prognosis. However, approximately 10–20% of children with PNS present with non-minimal change pathological variants, such as focal segmental glomerulosclerosis (FSGS). These cases are often steroid-resistant and are classified as steroid-resistant nephrotic syndrome (SRNS), which carries a poor treatment response and an unfavorable long-term outcome. It is estimated that 36–50% of children with SRNS experience recurrent disease progression within 10 years, ultimately leading to glomerulosclerosis and end-stage renal disease (ESRD)^1^.

Podocytes—specialized, terminally differentiated cells on the glomerular basement membrane (within Bowman’s capsule)—have interdigitating foot processes anchored to the membrane, with filtration slits linked by slit diaphragm proteins^2^. This structure forms the glomerular filtration barrier’s molecular sieve, blocking medium/high-molecular-weight proteins^3^. In FSGS, podocyte foot process/slit diaphragm injury causes massive proteinuria and progressive glomerulosclerosis^4^. Our study previously showed that podocyte injury accelerates adriamycin (ADR)-induced nephropathy in mice models^5^. Building on this,we have further investigated the mechanisms through which podocyte injury drives nephrotic syndrome progression to glomerulosclerosis.

CXC chemokine ligand 16 (CXCL16) is a multifunctional chemokine that exists in both transmembrane and soluble forms in humans^6,7^. Acting as a scavenger receptor, CXCL16 mediates the uptake and internalization of oxidized low-density lipoprotein (ox-LDL) by podocytes, leading to intracellular lipid accumulation and subsequent podocyte injury^6^. Our group has provided evidence, both in vitro and in vivo, that CXCL16 functions as a scavenger receptor contributing to podocyte injury in PNS^5,8,9^. Nevertheless, the upstream mechanisms regulating CXCL16 activation remain incompletely understood.

The purinergic ligand-gated ion channel 7 receptor (P2X7R), an ATP-gated trimeric ion channel of the P2X family, plays a significant role in physiological and pathological processes^10^. Ubiquitously expressed in various tissues, P2X7R activates multiple intracellular signaling pathways to mediate immune responses, oxidative stress, neurotransmitter release, cell proliferation, and apoptosis. ATP released upon cellular damage activates P2X7R, inducing inflammatory cytokine secretion and initiating inflammatory responses. In end-stage renal disease patients, P2X7R promotes atherosclerosis by regulating CXCL16/CXCR6 pathway activation^11^.The Nod-like receptor protein 3 (NLRP3) inflammasome, a cytosolic pattern recognition receptor complex composed of NLRP3, ASC, CARD, and pro-caspase-1^12^, is a major mediator of P2X7R-driven pathogenesis. Damage-induced ATP release activates P2X7R, triggering potassium channel opening and efflux; reduced intracellular potassium activates the NLRP3 inflammasome, which catalyzes the maturation of IL-1β and IL-18 precursors to drive inflammatory responses^13,14^. As the most characterized inflammasome, NLRP3 also exerts critical regulatory effects in various kidney diseases^15^. In our previous investigation using an ADR nephropathy mouse model, we demonstrated that elevated expression of P2X7R in renal tissues, accompanied by increased levels of NLRP3, IL-1β, and IL-18. Treatment with a P2X7R antagonist effectively downregulated their expression and reduced proteinuria, suggesting the potential involvement of the P2X7R/NLRP3 inflammasome pathway in the development and progression of ADR nephropathy^16^.

IL-18, belonging to the IL-1 cytokine superfamily, serves as an important immunomodulator. Clinical studies have documented significant alterations in IL-18 levels in children with nephrotic syndrome (NS) before and after treatment^17,18^. Furthermore, investigations into serum interleukin profiles in pediatric NS patients have revealed markedly elevated IL-1β concentrations in steroid-sensitive nephrotic syndrome (SSNS), indicating its potential utility as a biomarker for predicting steroid responsiveness^19^. In diabetic nephropathy research, IL-1β has been shown to induce tubulointerstitial damage through activation of the CXCL16 pathway^20^.

Based on these findings, we hypothesize that P2X7R may regulate downstream CXCL16 activation via the NLRP3 inflammasome pathway, ultimately contributing to podocyte injury and glomerulosclerosis in PNS. This study aims to investigate the effects and underlying mechanisms of P2X7R and NLRP3 inhibition on podocyte injury in PNS.

## Results

### Enhanced expression of P2X7R, NLRP3 and CXCL16 proteins in renal tissues of children with primary nephrotic syndrome

**Fig. 1:**
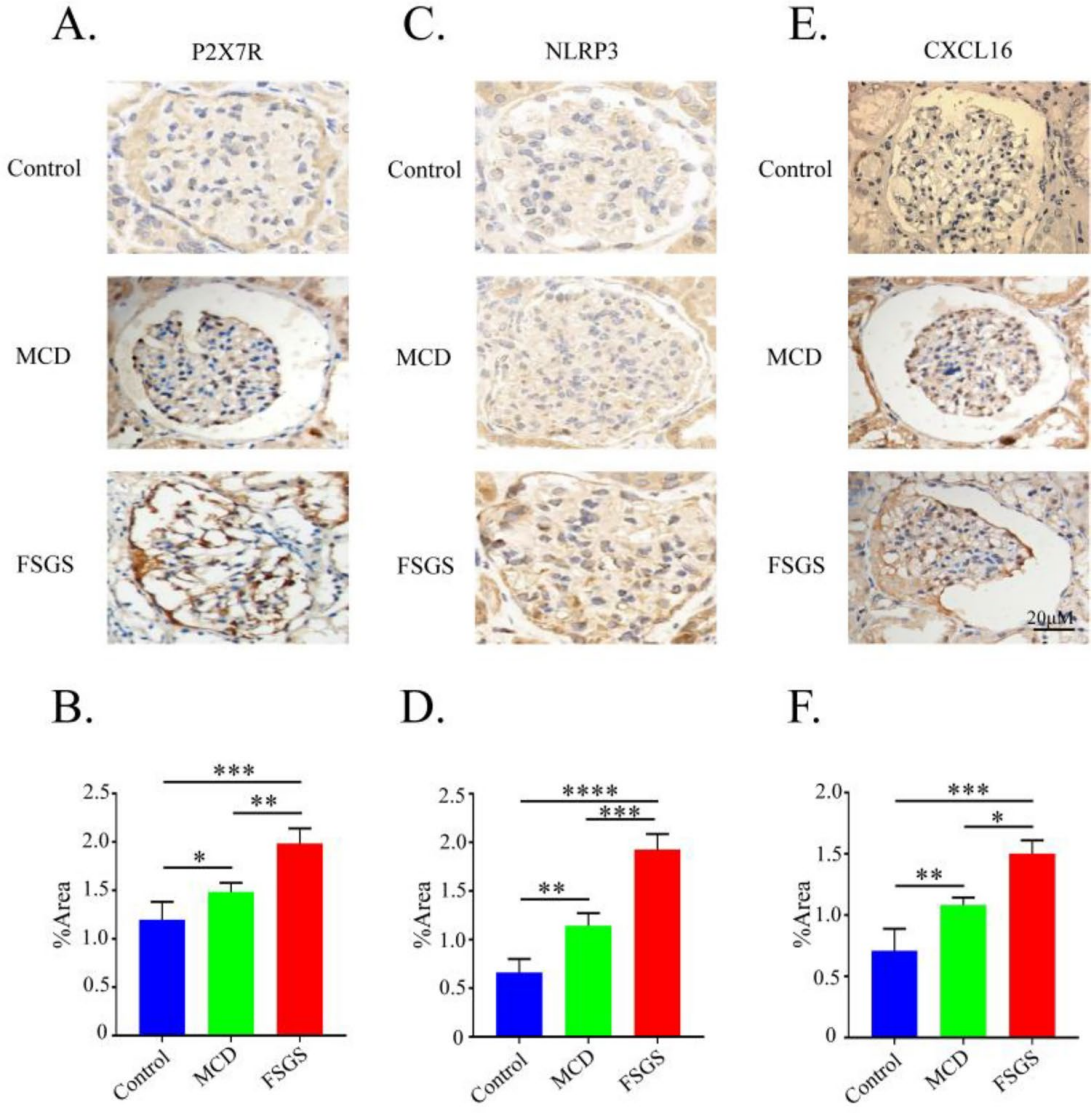
Expression of P2X7R, NLRP3 and CXCL16 in 15 cases of MCD and 15 cases of FSGS. Immunohistochemical staining of P2X7R (**A**), NLRP3 (**C**) and CXCL16 (**E**) in normal tissue (original magnification, ×200), MCD and FSGS. (**B**, **D** and **F**) Statistical results of gray scanning quantization in immunohistochemistry. Arrows represent podocytes. P2X7R, Purinergic Receptor P2X 7; CXCL16, CXC motif chemokine ligand 16; MCD, minimal change disease; FSGS, focal segmental glomerulosclerosis; *p < 0.05; **p < 0.01; ***p < 0.001; ****p < 0.0001.

Immunohistochemical analysis showed low expression of P2X7R, NLRP3, and CXCL16 in renal tissues of the normal control (NC) group, whereas significant upregulation was observed in tissues from children with PNS. Semi-quantitative analysis confirmed markedly higher expression levels of all three proteins in both MCD and FSGS compared to the NC group (*P* < 0.05). Moreover, the elevation of all three proteins was more pronounced in FSGS than in MCD (*P* < 0.05). All differences were statistically significant (Fig. 1).

### Effects of P2X7R knockdown on NLRP3, CXCL16, and desmin expression in ADR-induced podocyte injury

**Fig. 2:**
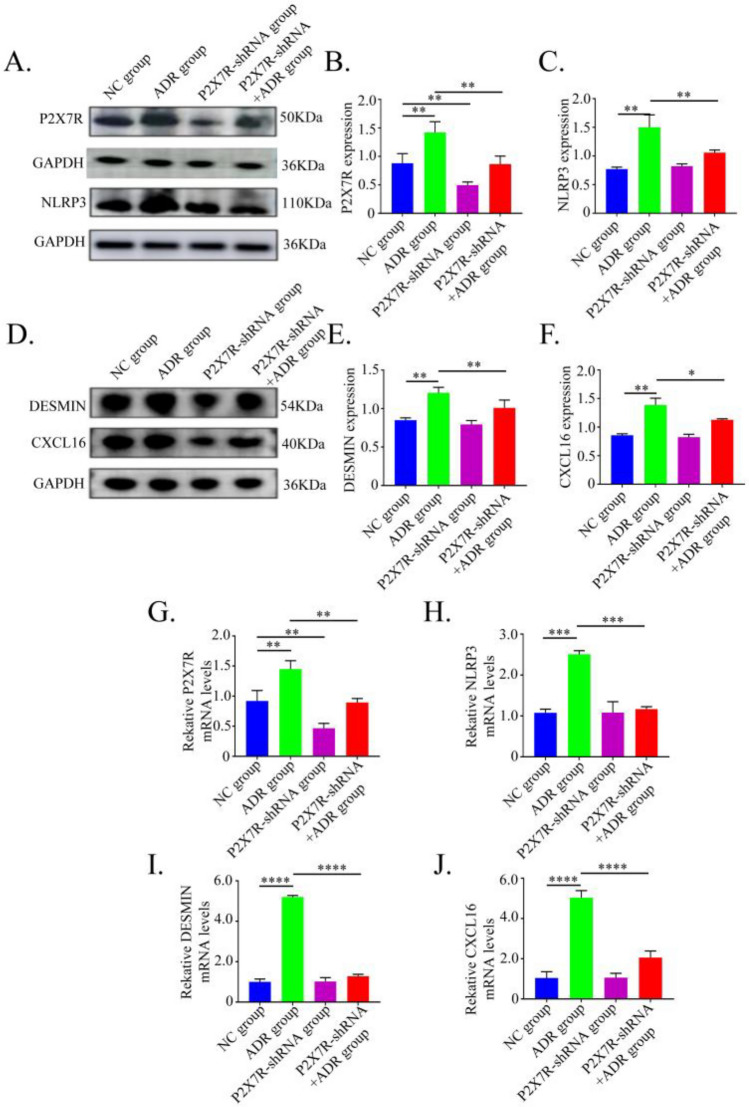
Expression of P2X7R, NLRP3, DESMIN and CXCL16 in HPC. (**A** and **D**) P2X7R, NLRP3, DESMIN and CXCL16 protein expression in HPC cell lines with P2X7R knockdown detected by western blotting. (**B**, **C**, **E** and **F**) Statistical results of gray scanning quantization in western blotting. (**G**–**J**) P2X7R, NLRP3, DESMIN and CXCL16 mRNA expression levels were evaluated by RT-qPCR in HPC. P2X7R, Purinergic Receptor P2X 7; CXCL16, CXC motif chemokine ligand 16; *p < 0.05; **p < 0.01; ***p < 0.001; ****p < 0.0001.

To test our hypothesis, we first knocked down P2X7R in podocytes using lentiviral transfection. Knockdown efficiency and cell proliferation activity were subsequently evaluated. RT-qPCR results confirmed a significant reduction in P2X7R mRNA expression following transfection, and CCK-8 assay showed no effect on cell proliferation.We then assessed relevant markers in human podocytes across treatment groups using Western blot and qPCR. Compared with the NC group, the ADR group exhibited significantly elevated protein and mRNA expression levels of P2X7R, NLRP3, CXCL16, and Desmin (*P* < 0.05). In contrast, these increases were markedly attenuated in the ADR + P2X7R-sh group relative to the ADR group (*P* < 0.05). No significant differences in the expression of NLRP3, CXCL16, or Desmin were observed between the NC and P2X7R-sh groups (*P* > 0.05) (Fig. 2).

### Effects of P2X7R knockdown on IL-1β and IL-18 expression in ADR-induced podocyte injury

**Fig. 3:**
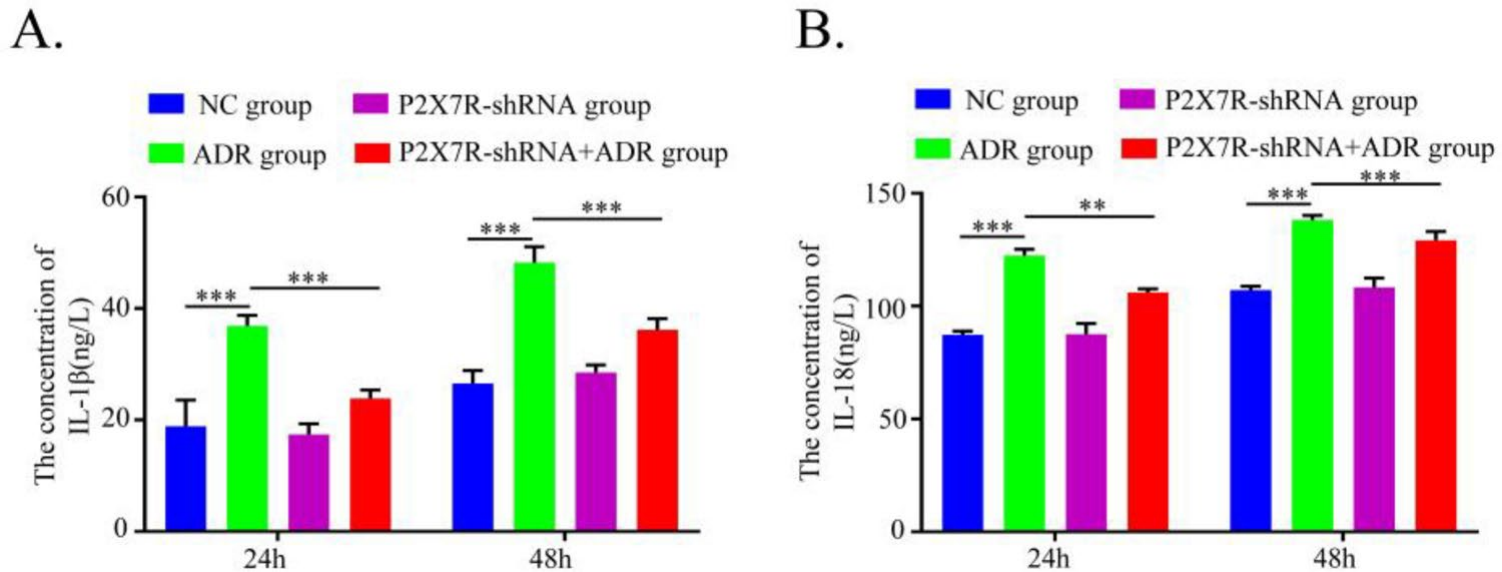
Levels of IL-1β and IL-18 in podocyte supernatant of each group after 24 h and 48 h. (**A**) The level of IL-1β was detected in each group. (**B**) The level of IL-18 was detected in each group. *P < 0.05; ***P < 0.001. P2X7R, Purinergic Receptor P2X 7; ADR, adriamycin. *p < 0.05; **p < 0.01; ***p < 0.001; ****p < 0.0001.

The levels of IL-1β and IL-18 in podocyte supernatant were measured by ELISA. Compared with the NC group, the ADR group showed significantly elevated expression of both inflammatory factors (*P* < 0.05). In contrast, knockdown of P2X7R (ADR + P2X7R-sh group) markedly reduced the levels of IL-1β and IL-18 relative to the ADR group (*P* < 0.05). No significant difference was observed between the NC group and the ADR + P2X7R-sh group (*P* > 0.05) (Fig. 3).

### Upregulation of NLRP3 in renal tissue of ADR-induced nephropathy mice

**Fig. 4:**
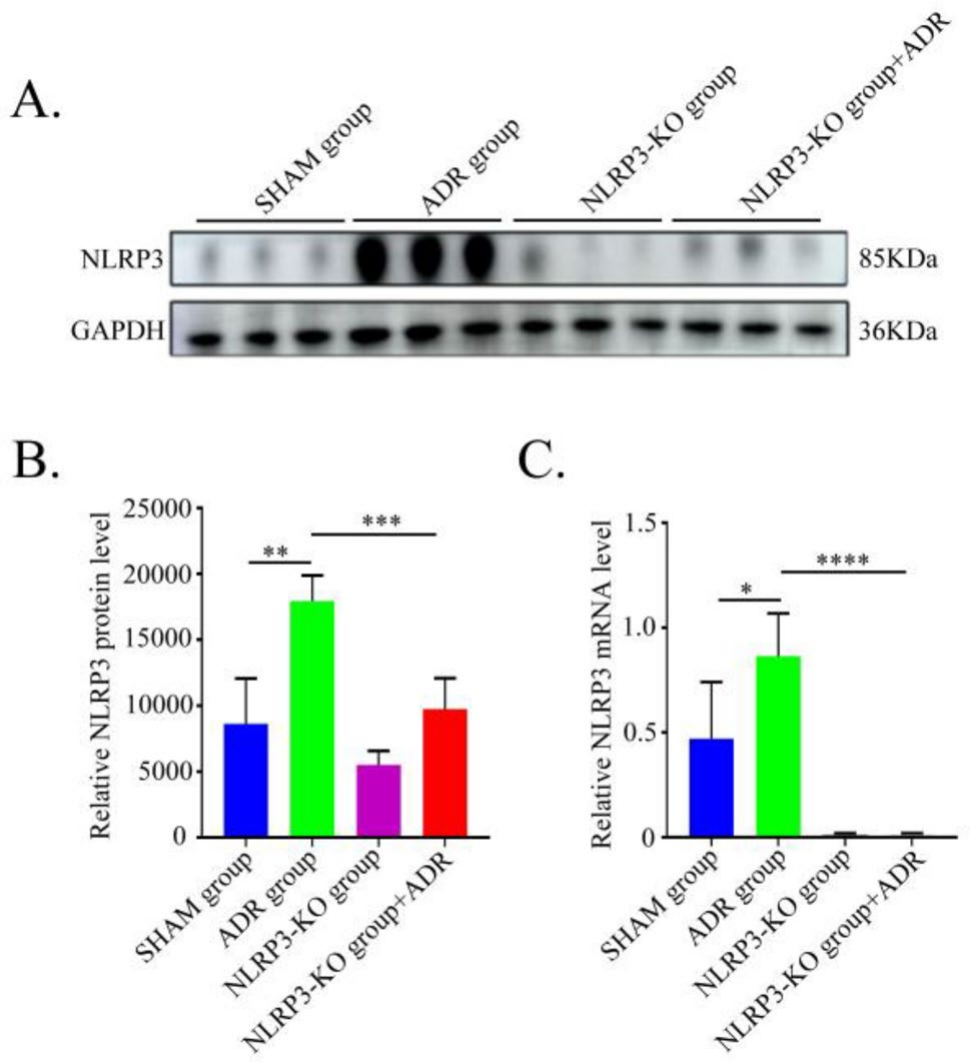
Expression of NLRP3 in each group (n = 6). (**A**) NLRP3 protein expression in mice with NLRP3 knockdown or/and ADR treatment detected by western blotting. (**B**) Statistical results of gray scanning quantization in western blotting. (**C**) nlrp3 expression levels were evaluated by RT-qPCR in each group. *P < 0.05; **P < 0.01; ***P < 0.001.

To investigate the role of NLRP3 in podocyte injury in NS, we established ADR-induced nephropathy models in NLRP3 knockout (KO) mice and assessed subsequent pathological changes. Following the successful establishment of ADR nephropathy models and nlrp3 gene knockout, we assessed NLRP3 expression at both protein and transcriptional levels in renal tissues of different groups using Western Blot and RT-qPCR. The results demonstrated that NLRP3 protein and mRNA expression were significantly elevated in the ADR nephropathy group compared with the NC group (*P* < 0.05). In contrast, NLRP3 knockout markedly attenuated these increases, showing substantially reduced protein and mRNA expression levels in the kidney of ADR-induced nephropathy mice (*P* < 0.05) (Fig. 4).

### NLRP3 knockout ameliorates urinary and biochemical indicators in ADR-induced nephropathy mice

**Fig. 5:**
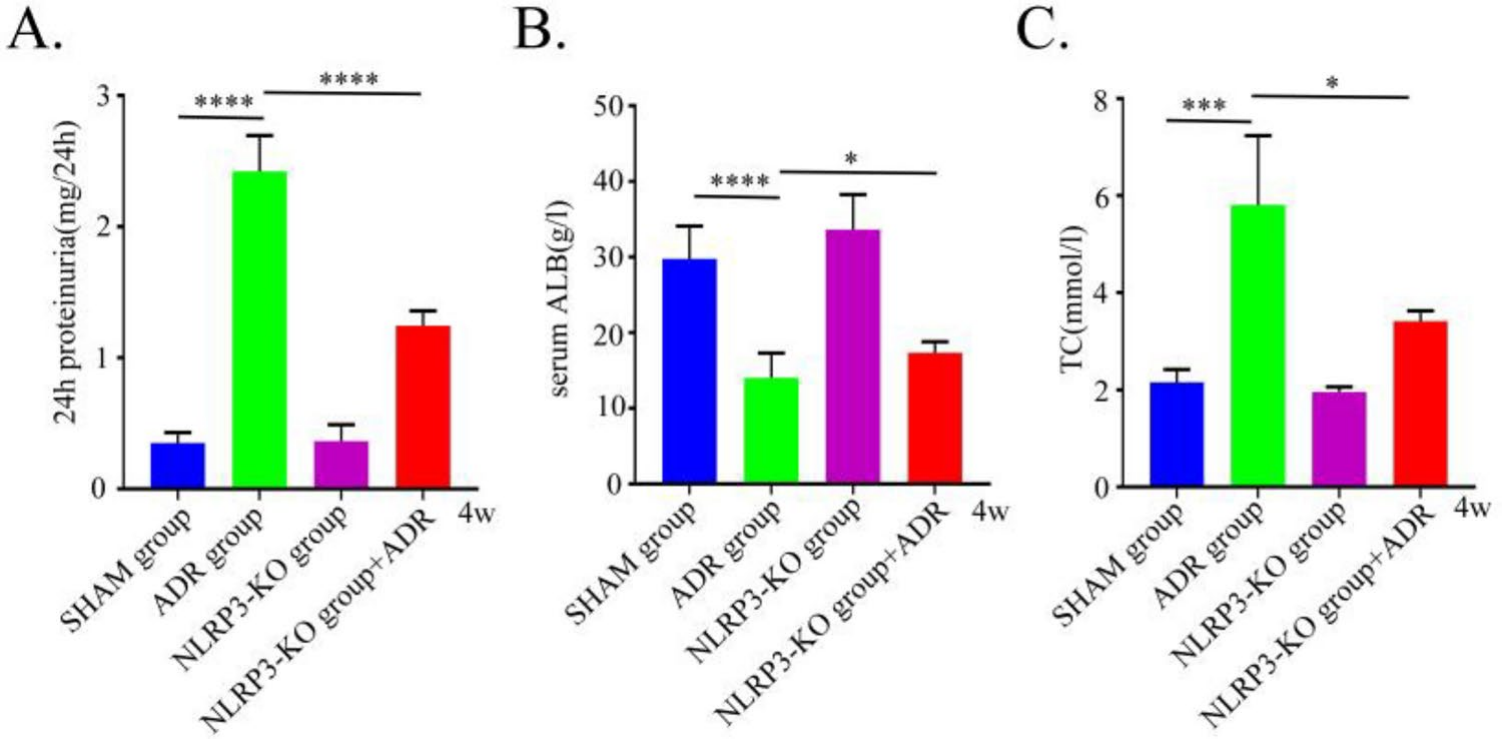
Evaluation of NS in mice with ADR-induced nephropathy or/and NLRP3 knockout (n = 6). (**A**–**C**) Measurement of 24 h urinary protein, serum albumin, and serum total cholesterol levels in mice from each group. SHAM, control group; ADR 4W, ADR-induced nephropathy model group at 4 weeks. *P < 0.05; ***P < 0.001; ****P < 0.0001.

We evaluated the effect of NLRP3 knockout on key disease indicators in ADR-induced nephropathy mice. Compared with the ADR nephropathy group, NLRP3 knockout resulted in a significant reduction in 24 h urine protein (*P* < 0.0001), a marked increase in serum albumin (*P* < 0.05), and a significant decrease in total serum cholesterol (*P* < 0.05) (Fig. 5).

### NLRP3 knockout attenuates podocyte injury in ADR-induced nephropathy mice

**Fig. 6:**
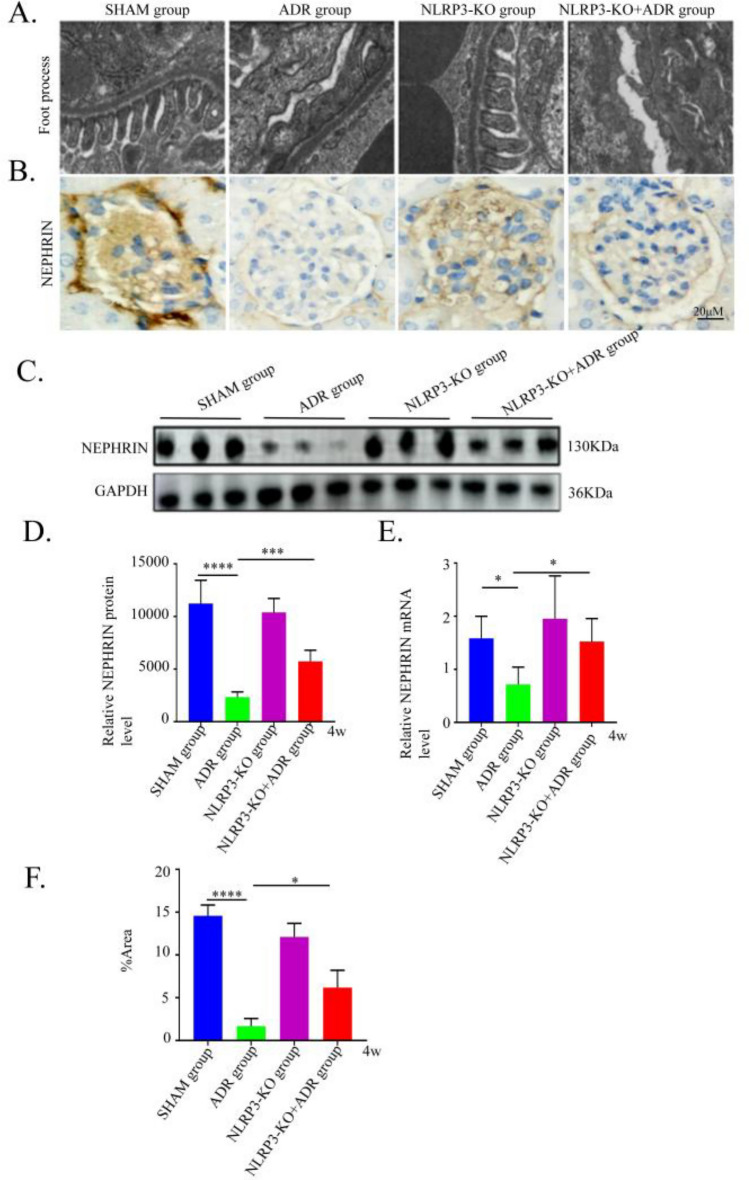
Evaluation of podocyte injury and NEPHRIN expression in each group (n = 6). (**A**) Electron microscopy images showing the podocyte foot process morphology in each group (× 15 k; scale bar, 500 nm). (**B**) Immunohistochemical staining of NEPHRIN in each group (original magnification, ×200). (**C**) NEPHRIN protein expression in each group detected by western blotting. (**D**) Statistical results of gray scanning quantization in western blotting. (**E**) NEPHRIN expression levels were evaluated by RT-qPCR in each group. (**F**) The semi-quantitative results of NEPHRIN immunohistochemical staining in each group. SHAM, control group; ADR4W,ADR-induced nephropathy model group at 4 weeks. *P < 0.05; ***P < 0.001; ****P < 0.0001.

Podocyte morphology was examined by TEM. In the SHAM group, the glomerular basement membrane was uniform, and podocytes with intact foot processes were observed. In contrast, the ADR nephropathy group exhibited extensive foot process effacement. NLRP3 knockout alleviated this injury, showing only partial foot process fusion (Fig. 6A).

We further assessed Nephrin, a key slit diaphragm protein and podocyte biomarker, using Western blot, immunohistochemistry, and RT-qPCR. Both protein and mRNA expression levels of Nephrin were significantly downregulated in ADR nephropathy mice compared with the SHAM group (*P* < 0.05). However, NLRP3 knockout significantly restored Nephrin expression at both transcriptional and protein levels (*P* < 0.05) (Fig. 6B-E).

### Effect of NLRP3 knockout on CXCL16 expression in ADR-induced nephropathy mice

**Fig. 7:**
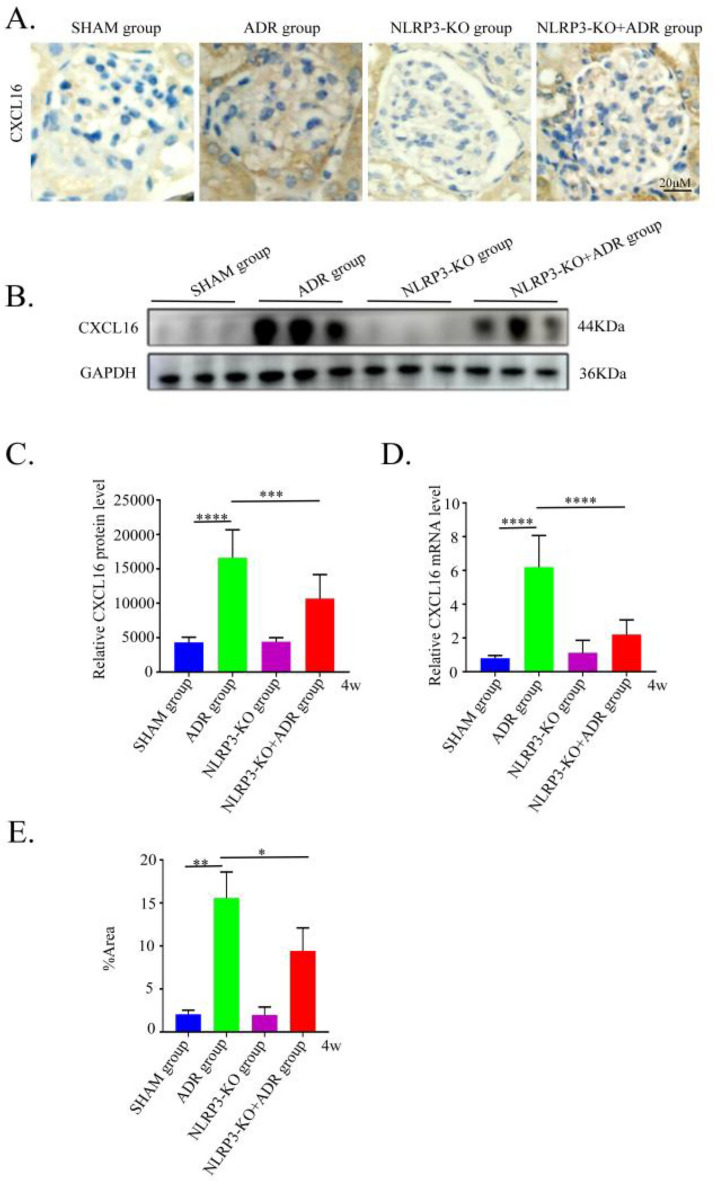
Expression of CXCL16 in each group (n = 6). (**A**) Immunohistochemical staining of CXCL16 in each group (original magnification, ×200). (**B**) CXCL16 protein expression in each group detected by western blotting. (**C**) Statistical results of gray scanning quantization in western blotting. (**D**) CXCL16 expression levels were evaluated by RT-qPCR in each group. (**E**) The semi-quantitative results of CXCL16 immunohistochemical staining in each group. SHAM, control group; ADR4W, ADR-induced nephropathy model group at 4 weeks. ***P < 0.001; ****P < 0.0001.

To investigate whether CXCL16 expression is regulated by NLRP3 in ADR-induced nephropathy, we assessed CXCL16 expression at both protein and mRNA levels in renal tissues using Western blot, immunohistochemistry, and RT-qPCR. Compared with the SHAM group, CXCL16 expression was significantly upregulated in ADR-induced nephropathy mice (*P* < 0.05). However, genetic knockout of NLRP3 markedly downregulated both CXCL16 protein and mRNA expression in the renal tissues of ADR-induced nephropathy mice (*P* < 0.05) (Fig. 7).

### Effects of NLRP3 knockout on IL-18 and IL-1β expression in ADR-induced nephropathy mice

**Fig. 8:**
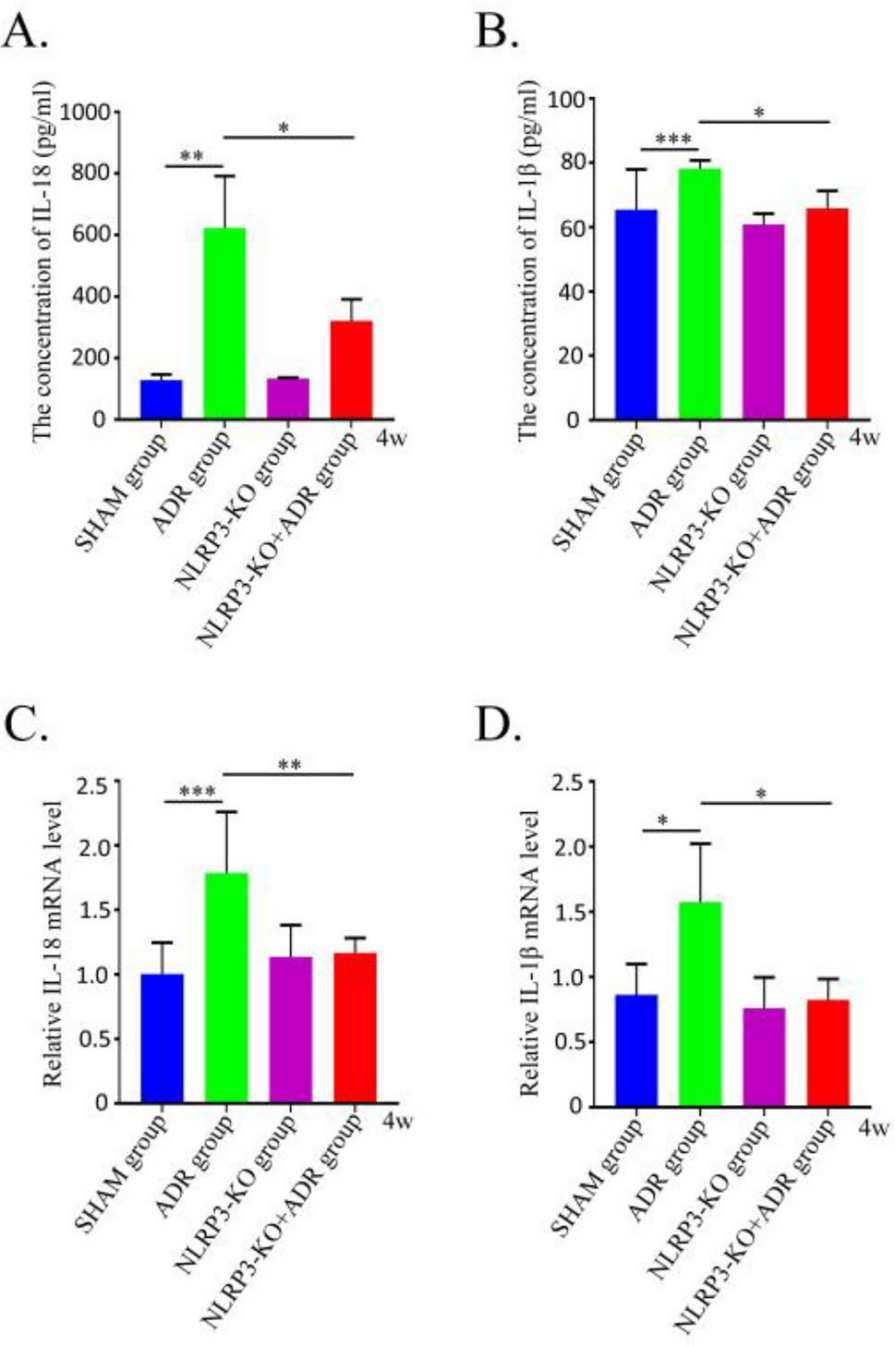
Levels of IL-1β and IL-18 in each group (n = 6). (**A**) The serum level of IL-18 was detected in each group. (**B**) The serum level of IL-1β was detected in each group. (**C**) IL-18 expression levels were evaluated by RT-qPCR in each group. (**D**) IL-1β expression levels were evaluated by RT-qPCR in each group. *P < 0.05; **P < 0.01; ***P < 0.001.

The expression levels of the inflammatory factors IL-18 and IL-1β in mouse serum and renal tissues were assessed by ELISA and RT-qPCR, respectively. Compared with the SHAM group, both IL-18 and IL-1β levels were significantly elevated in the ADR-induced nephropathy group (*P* < 0.05). However, knockout of the NLRP3 gene markedly reduced the expression of these inflammatory factors in both serum and renal tissues of ADR-induced nephropathy mice (*P* < 0.05) (Fig. 8).

**Fig. 9:**
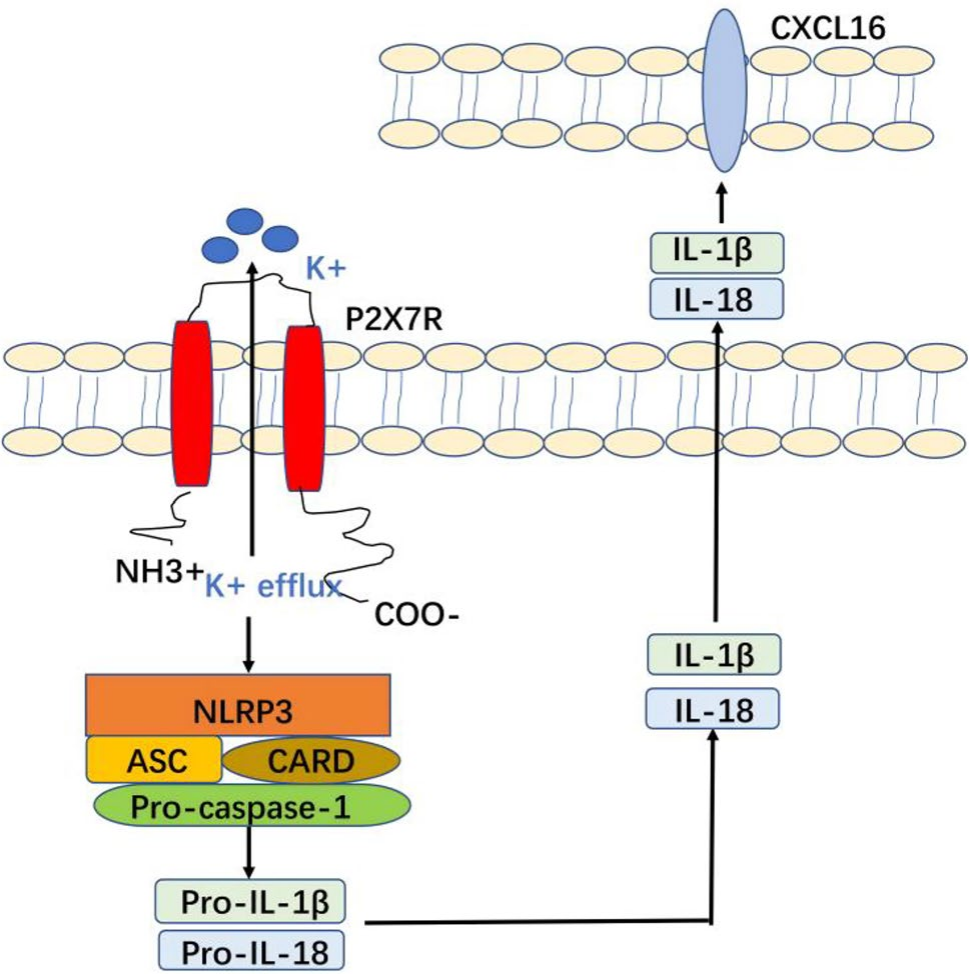
Mechanistic diagram of the P2X7R-NLRP 3-IL-1β pathway regulates CXCL16 in podocyte injury.

Based on the above results, the potential mechanism underlying podocyte injury induced by the P2X7R-NLRP3-IL-1β pathway regulating CXCL16 is summarized in (Fig. 9). Our data demonstrate that activation of P2X7R triggers K⁺efflux, which subsequently assembles the NLRP3 inflammasome complex. This complex promotes the cleavage of pro-IL-1β and pro-IL-18 into their mature forms, ultimately inducing the upregulation of CXCL16 and subsequent podocyte injury.

## Discussion

PNS is a glomerular disorder characterized by massive proteinuria (> 3.5 g/24 h), hypoalbuminemia (serum albumin < 30 g/L), hyperli-pidemia, and significant edema. It is particularly prevalent in children, among whom MCD represents the predominant pathological subtype, accounting for approximately 80–90% of children with PNS. These patients generally respond well to steroid therapy and have a favorable prognosis. However, in 10–20% of pediatric cases, non‑minimal change pathology—such as FSGS—is present, which is often steroid-resistant and associated with poorer treatment outcomes^21,22^. Studies indicate that 36–50% of children with SRNS experience recurrent disease progression within 10 years, leading to glomerulosclerosis and ESRD^1^. Therefore, elucidating the pathogenesis of PNS and identifying key therapeutic targets have become urgent priorities in the field.

Podocytes, terminally differentiated cells on the outer surface of the glomerular basement membrane, possess foot processes apposed to the membrane and filtration slits sealed by slit diaphragm proteins^2^. As a core component of the glomerular filtration barrier, they prevent leakage of medium and high molecular weight proteins^3^. Current evidence classifies MCD and FSGS as podocytopathies characterized by graded podocyte injury^23^. In FSGS, aberrations in podocyte foot processes and slit diaphragms induce massive proteinuria and subsequent glomerulosclerosis^4^. Podocyte injury downregulates key proteins (Nephrin, Podocin, tight junction protein ZO-1) and upregulates Desmin^24,25^. The ADR-induced nephropathy model recapitulates human nephrotic syndrome, exhibiting early MCD-like and late FSGS-like pathology^26,27^. Our prior work demonstrated that podocyte injury drives the development and progression of ADR nephropathy^5^; herein, we utilize in vitro ADR-induced podocyte injury and in vivo ADR nephropathy mouse models to investigate the specific mechanisms underlying podocyte injury in nephrotic syndrome.

CXCL16 is a dual-form chemokine (transmembrane and soluble) with multifunctional roles as both a scavenger receptor and a chemokine in humans. Glomerular podocytes express CXCL16, which mediates the uptake of ox-LDL, resulting in intracellular lipid accumulation and subsequent podocyte injury^6^. Clinically, CXCL16 levels in children with active PNS are positively correlated with blood lipids and 24-h urine protein, and negatively correlated with serum albumin, implying its involvement in PNS pathogenesis^8^. Animal experiments further confirmed that in ADR-induced nephropathy, CXCL16 acts as a scavenger receptor for ox-LDL to facilitate its podoctye uptake, thereby promoting podocyte injury and glomerulosclerosis^5^. However, the upstream regulatory mechanisms underlying CXCL16-mediated podocyte damage remain elusive .

P2X7R, a member of the P2X receptor family, is an ATP-gated trimeric ion channel that is widely expressed in various tissues^28^. It activates multiple intracellular signaling pathways and participates in diverse physiological processes (e.g., immune response, oxidative stress). Upon cellular damage, extracellular ATP activates P2X7R, inducing the release of proinflammatory cytokines (e.g., IL-1β) and triggering inflammatory responses. Given the roles of inflammation and immunity in numerous clinical diseases, P2X7R is implicated in the pathogenesis of various inflammatory conditions^11,29^. Previous studies have reported upregulated P2X7R expression in disease models linked to cellular injury and inflammation, including hypertension, type 1 diabetes and acute glomerulonephritis^30^. Additionally, in patients with ESRD, P2X7R may promote atherosclerosis by regulating the CXCL16/CXCR6 pathway^11^. In vitro studies, we observed increased P2X7R expression in human podocytes treated with ox-LDL, suggesting its involvement in ox-LDL-induced podocyte injury^31^. To further explore the role of P2X7R in ADR-induced podocyte injury, we used lentivirus-mediated P2X7R knockdown. This study first confirmed that P2X7R knockdown did not affect podocyte viability. We then demonstrated that P2X7R expression was upregulated in ADR-injured podocytes, accompanied by increased intracellular Desmin expression; notably, P2X7R knockdown significantly reduced Desmin expression. These findings indicate that P2X7R contributes to ADR-induced podocyte injury. However, whether P2X7R acts as an upstream regulator of CXCL16 activation and thereby promotes glomerulosclerosis in podocyte injury-related nephropathy. And the specific underlying mechanisms remain unreported in the literature.

The NLRP3 inflammasome, a core component of the NLR family, consists of NLRP3, ASC, CARD, and the effector protein pro-caspase-1. As a critical immune sensor, it recognizes signals from exogenous pathogens and endogenous cellular damage, initiating inflammasome assembly to mediate downstream responses. Among its regulatory pathways, the P2X7R/NLRP3 axis is one of the most well-characterized and is linked to the pathogenesis of multiple diseases^32,33^. Following cellular injury, extracellular ATP activates P2X7R, reducing intracellular potassium levels and thereby triggering NLRP3 inflammasome activation. This process drives cleavage of pro-IL-1β and pro-IL-18 into their mature forms (IL-1βand IL-18), ultimately promoting inflammatory responses^13,14^. Furthermore, accumulating evidence implicates inflammatory cytokines in PNS. IL-18, a member of the IL-1 cytokine superfamily, acts as a key modulator of immune responses, and its levels show significant alterations in children with PNS both pre- and post-treatment^17,18^. Additionally, serum IL-1β is markedly elevated in SSNS, and may serve as a reliable biomarker for predicting steroid therapy responses^19^. Recent studies on diabetic nephropathy further demonstrate that IL-1β induces tubulointerstitial injury via activation of the CXCL16 pathway^20^.

To investigate whether the P2X7R-NLRP3-IL-1β axis contributes to podocyte injury in PNS by activating CXCL16, we first assessed the expression of P2X7R, NLRP3, and CXCL16 in renal tissues from pediatric NS patients using immunohistochemistry. Consistent with our previous findings^34^, glomerular expressions of P2X7R, NLRP3, and CXCL16 were elevated in both MCD and FSGS, with more pronounced upregulation in FSGS. This suggests that these molecules may collectively contribute to PNS progression.

In earlier studies using ADR nephropathy models, we observed significant upregulation of NLRP3, CXCL16, IL-1β, and IL-18 in renal tissues, which was attenuated upon P2X7R inhibition. These results imply that the P2X7R/NLRP3 pathway may regulate CXCL16 during the development of ADR-induced kidney injury^16^. In the present study, we aimed to examine in vitro whether the P2X7R/NLRP3 pathway mediates ADR-induced podocyte injury via CXCL16. In an ADR-induced podocyte injury model, expressions of P2X7R, NLRP3, CXCL16, IL-1β, and IL-18 were markedly increased. However, knockdown of P2X7R significantly suppressed their expression. So we speculate that P2X7R/NLRP3 may indirectly regulate CXCL16 through downstream inflammatory factors such as IL-1β in ADR-induced podocyte injury.

Numerous prior studies have demonstrated that inflammasome activation is implicated in acute kidney injury, fibrosis, and chronic kidney disease, ultimately leading to irreversible renal damage^35^. In particular, the NLRP3 inflammasome has been shown to contribute to the pathogenesis of various kidney diseases^36^. For instance, long-term high-fat diet intake in experimental animals can induce obesity-related glomerulopathy, accompanied by significant upregulation of NLRP3 inflammasome expression in podocytes, suggesting its role in promoting podocyte injury under obese conditions^37^. In diabetic nephropathy, NLRP3 inhibition—either by genetic knockout or pharmacological means—has been shown to mitigate lipid accumulation, protect against podocyte injury, and attenuate renal inflammation and fibrosis^38^. However, the role and underlying mechanisms of NLRP3 in podocyte injury in PNS remain poorly understood.

In this study, we employed the ADR-induced nephropathy model to investigate the effect of NLRP3 knockout on podocyte injury. As successful established the ADR nephropathy model and NLRP3 knockout, we found that NLRP3 knockout significantly ameliorated clinical parameters in ADR nephropathy mice. In addition, the expression of the podocyte marker Nephrin was restored, and electron microscopy revealed improved podocyte morphology, collectively indicating the involvement of NLRP3 in ADR-induced podocyte injury. To further elucidate the underlying mechanism, we examined related molecular pathways. Consistent with our earlier report^16^, ADR nephropathy mice exhibited elevated renal levels of NLRP3, CXCL16, and the inflammatory cytokines IL-1β and IL-18. However, these increases were markedly suppressed in NLRP3-knockout ADR-induced mice, suggesting that the NLRP3 inflammasome contributes to podocyte injury, at least in part, through regulation of CXCL16 and associated inflammatory mediators.

In conclusion, our study is the first to demonstrate the role of the P2X7R-NLRP3-CXCL16 axis in podocyte injury of primary nephrotic syndrome. This finding provides novel theoretical insights and identifies P2X7R and NLRP3 as promising therapeutic targets for clinical translation. Collectively, our work highlights the potential of targeting this axis as a strategy for primary nephrotic syndrome and outlines key future directions, including validating our findings in human samples and exploring combinatorial therapeutic approaches.

Notably, our current study focused on the direct link between P2X7R/NLRP3 and CXCL16 in podocyte injury, and we did not perform large-scale omics analyses to globally map the downstream pathways of P2X7R/NLRP3. This represents a limitation of our work, as omics approaches (e.g., transcriptomics, proteomics) could provide a more comprehensive view of the regulatory network. However, our findings clearly demonstrate that P2X7R/NLRP3 inhibition effectively mitigates podocyte injury and suppresses CXCL16 expression, which aligns with the known role of P2X7R/NLRP3 in driving inflammatory responses. In future studies, we plan to conduct comprehensive omics analyses to systematically identify and validate all downstream pathways of P2X7R/NLRP3 in this context, which will further deepen our understanding of the molecular mechanisms underlying podocyte injury.

## Materials and methods

### Cell culture

Immortalized human podocytes (HPCs), kindly provided by Professor Rong Wang from the Department of Nephrology at Shandong Provincial Hospital Affiliated to Shandong University, were used for in vitro culture. The immortalized podocyte cell line was initially cultured in a 33 °C in complete medium, which consisted of RPMI-1640 medium supplemented with 10% fetal bovine serum and penicillin–streptomycin. After approximately 48 h, the cells were transferred to a 37 °C incubator to promote growth and differentiation. The podocytes were allowed to mature for at least two weeks until a fully differentiated state was achieved.

### Lentivirus transfection of podocytes

HPC cells were seeded in 6-well plates at a density of (3–5) × 10^5^ cells per well and cultured overnight at 37 ℃. The next day, the medium was replaced with polybrene-containing medium. To optimize transduction conditions, a lentivirus multiplicity of infection (MOI) gradient (5, 10, 30, 50, 100) was set up in 96-well plates; the optimal MOI was defined as the value yielding ~ 80% green fluorescent podocytes. When cell confluency reached 30–50%, transduction was performed at the pre-determined optimal MOI. Transduction efficiency was evaluated via fluorescence microscopy at 48–72 h post-transduction. Stable cell lines were selected with puromycin, after which total protein and RNA were extracted from negative control and P2X7R-knockdown groups. P2X7R knockdown efficiency was validated at the protein (Western blot) and mRNA (qPCR) levels, respectively.

### Cell grouping

HPC cells were randomly divided into four groups: (1) NC group: Cells were transfected with negative control lentivirus for 72 h only; (2) ADR group: After 72 h of negative control lentivirus transfection, cells were stimulated with 1 μmol/mL ADR for induction; (3) P2X7R-shRNA group: Following 72 h of lentivirus‐mediated P2X7R-shRNA transfection, cells were selected with puromycin for 24-48 h to establish a stable cell line; (4) ADR + P2X7R shRNA group: After 72 h of lentivirus transfection (P2X7R‑shRNA), a puromycin‐selected stable cell line was treated with 1 μmol/mL ADR for 24 h to induce injury.

### Cell proliferation

Cell proliferation activity was assessed in both the blank control group and the P2X7R-shRNA group. HPC cells were seeded into 96-well plates at a density of 1 × 10^4^ cells per well, with five replicate wells per group, and incubated at 37 °C under 5% CO_2_ for 24 h. After the initial incubation, one plate was randomly selected, and 10 µL of CCK-8 solution was added to each well, followed by additional incubation for 2 h. The optical density (OD) at 450 nm was then measured using a microplate reader. The same procedure was repeated with the remaining two plates after 48 and 72 h of culture, respectively, to determine OD values at these time points.

### Experimental animals

C57BL/6J-NLRP3-KO male mice and their wild-type C57BL/6J male littermates (5 weeks old, body weight 18–22 g) were obtained from Shanghai Southern Model Biotechnology Co., LTD. All mice were housed under specific pathogen-free (SPF) conditions with a 12 h light/dark cycle, ambient temperature maintained at 20–24 °C, and ad libitum access to food and water for 7 days. All animal experiments were conducted in accordance with the ARRIVE 2.0 guidelines (https://arriveguidelines.org) and were approved by the Ethics Committee of Clinical Medical College of Shandong University (Approval No.:SDULCLL2021-2-32).

### Establishment and grouping of ADR nephropathy mouse model

C57BL/6J young male mice were randomly assigned to four groups (n = 10 per group): a sham-treated control group (SHAM group), an ADR-induced nephropathy model group (ADR group), an NLRP3 knockout group (NLRP3-KO group), and an NLRP3 knockout group subjected to adriamycin treatment (NLRP3-KO + ADR group). Mice in the ADR and NLRP3-KO + ADR groups received a single tail vein injection of adriamycin (25 mg/kg), whereas those in the SHAM and NLRP3-KO groups were administered an equivalent volume of saline via the same route. Mice that died during modeling were excluded, and 6 successfully modeled mice were randomly selected from the survivors in each group for subsequent experiments. At 4 weeks post-injection, following a 24 h urine collection, all the mice were anesthetized by intraperitoneal of sodium pentobarbital (100 mg/kg) and then euthanized by an overdose of intraperitoneal of sodium pentobarbital. Death was confirmed by lack of response to stimuli. Serum, 24 h urine samples, and kidney tissues were collected for subsequent analysis. A portion of each kidney tissue sample was fixed in 4% paraformaldehyde for immunohistochemical evaluation, while another portion was immersed in 2.5% glutaraldehyde for ultrastructural observation via scanning electron microscopy. The remaining kidney tissues and serum samples were stored at − 80 °C for future mRNA and protein analyses.

### Enzyme-linked immunosorbent assay

Concentrations of IL-1β and IL-18 in HPC cell supernatants and mouse serum were quantified via commercial ELISA kits following the manufacturer’s protocols. Briefly, cell culture supernatants from each experimental group were collected, and standards/samples were diluted with the kit-supplied dilution buffer. A 96-well plate was prepared with blank control, standard, and sample wells, after which 50 μl of standard or diluted sample was added per well. The plate was sealed and incubated at 37 °C for 30 min. Following PBS washing, 50 μl of enzyme-conjugated detection antibody was added to all wells except blanks, and the plate was incubated again at 37 °C for 30 min before another PBS wash. Next, 50 μl of chromogen substrate A and 50 μl of substrate B were sequentially added to each well; the plate was gently shaken for mixing and incubated at 37 °C in the dark for 10 min. The reaction was terminated with stop solution, and absorbance was measured at 450 nm using a microplate reader. A standard curve was generated by plotting optical density values against standard concentrations.

### Western blotting

Podocytes and renal tissues were lysed using RIPA buffer. Renal tissues were homogenized on ice with an ultrasonic disruptor, and protein concentrations were determined with a BCA kit (Solarbio, Beijing, China). Proteins (20 µg per lane) were separated by 10% SDS-PAGE and transferred onto PVDF membranes. The membranes were incubated overnight at 4 °C with the following primary antibodies: rabbit anti-P2X7R (1:1000, ab307718, Abcam), anti-CXCL16 (1:1000, ab307694, Abcam; and 1:200, bs-1441R, Bioss), anti-NLRP3 (1:1000, a5652, ABclonal), anti-Nephrin (1:1000, ab216341, Abcam), anti-Desmin (1:100, ab227651, Abcam), and anti-GAPDH (1:5000, ab8245, Abcam). After washing, the membranes were incubated with corresponding HRP-conjugated secondary antibodies at room temperature for 2 h. Following another wash with TBST (Tris-buffered saline with 0.2% Tween-20), protein bands were visualized using a chemiluminescence detection system (BD Rad, USA) and analyzed quantitatively with Image J 6.0 software.

### Immunohistochemical analysis

The clinical renal tissue samples from children with nephrotic syndrome were obtained from anonymized, archived renal biopsy specimens at Shandong Provincial Hospital. This study was reviewed and approved by the Medical Ethics Committee of Clinical Medical College of Shandong University, which waived the requirement for informed consent due to the use of de-identified, leftover clinical materials (Ethics Approval No. SDULCLL2021-1-32).

Human and mouse kidney tissues were dehydrated through a graded ethanol series and embedded in paraffin. Sections of 5 µm thickness were cut, dewaxed in xylene, and rehydrated through a descending ethanol gradient. After washing with 0.1 M PBS, antigen retrieval was performed by boiling the sections in EDTA buffer (pH 8.0) at 100 °C for 5 min, followed by cooling at room temperature for 2 h. Endogenous peroxidase activity was blocked by treating the sections with 3% hydrogen peroxide for 30 min. The sections were then blocked with 10% goat serum for 1 h at room temperature and incubated overnight at 4 °C with the following primary antibodies: rabbit anti-CXCL16, rabbit anti-NLRP3, rabbit anti-P2X7R, and rabbit anti-Nephrin. After washing, the sections were incubated with a goat anti-rabbit secondary antibody for 30 min at room temperature. Color development was performed using DAB substrate, and counterstaining was carried out with hematoxylin. All specimens were examined under an optical microscope at 400× magnification.

### Quantitative real-time PCR

Total RNA was extracted from the cells with Trizol reagent (TaKaRa, Japan) according to the manufacturer’s instructions. Subsequently, RNA was reverse-transcribed into complementary DNA (cDNA) using the PrimeScriptTM reagent kit (TaKaRa, Japan). Quantitative real-time PCR (qRT-PCR) was then performed with the following primer sequences: P2X7R forward primer: 5′-GCCACAACTACACCACG AGAA-3′ and reverse primer: 5′-GCCCTGAATTGCCACATCTGA-3′; NLRP3 forward primer: 5′-GATCTTCGCTGCGATCAACA-3′ and reverse primer: 5′-GGGA TTCGAAACACGTGCATTA-3′; Desmin forward primer: 5′-CTCTGCCCTCAACT TCCGAG-3′ and reverse primer: 5′-CTTCATGCTGCTGTGTG-3′; CXCL16 forward primer: 5′-TCCCACAGCCAGGACATCAG-3′ and reverse primer:5′-AGGTATTAGT CAGGTGCCACA-3′; Nephrin forward primer: 5′-CTCTGCCCTCAACTTCCGAG-3′ and reverse primer: 5′-CTTCATGTGTGCTGTGTG-3′; IL-1β forward primer: 5′-ATGATGGCTTATTACAGTGGCAA-3′ and reverse primer: 5′-GTCGGAGATTC GTAGCTGGA-3′; IL-18 forward primer: 5′-CCCTCTCCCCAAGCTTACTT-3′ and reverse primer: 5′-TTCAGTGGAACAGGAGTCCA-3′; GAPDH forward primer: 5′-GCACCGTCAAGGCTTTGAGAAC-3′ and reverse primer: 5′-TGGTGAAGACG CAGTGGA-3′. qPCR was performed using the LightCycler® 480 system with SYBR Green master mix (Applied Biological Systems). Each sample was run in triplicate, and gene expression levels were normalized to GAPDH as the endogenous control. Relative quantification was calculated using the 2^(− ΔΔCT) method.

### Statistical analysis

Statistical analyses were performed using GraphPad Prism version 9.0. Continuous data, including the semi-quantitative %Area values derived from immunohistochemical staining, are presented as the mean ± standard deviation (SD). For comparisons among the four experimental groups, one-way analysis of variance (ANOVA) was used, followed by Tukey’s post hoc tests for pairwise comparisons between any two groups. All statistical tests were two-sided, and a *P*-value of less than 0.05 was considered statistically significant.

## Supplementary Information

Supplementary Information 1.

Supplementary Information 2.

## Data Availability

The datasets generated and/or analyzed in the present study are available from the corresponding author upon reasonable request.
