# Supplementary material for: Inhibiting the P2X7R-NLRP3 inflammasome pathway regulates CXCL16 to alleviate podocyte injury in mice with adriamycin nephropathy
Source: Sci Rep. 2026 Apr 5;16:16361. doi: 10.1038/s41598-026-47345-5 (PMC13212586; doi:10.1038/s41598-026-47345-5)
Supplement: Supplementary file 1 — Supplementary Information 1. [file 41598_2026_47345_MOESM1_ESM.pdf]

1.Full-length gel/blot for Figure 2

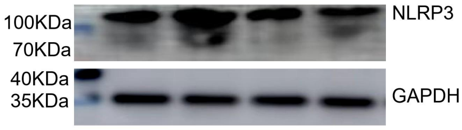

labeling：the grouping of gels/blots cropped from different parts of the same gel

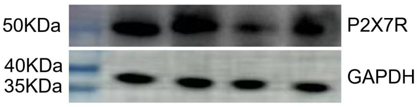

labeling：the grouping of gels/blots cropped from different parts of the same gel

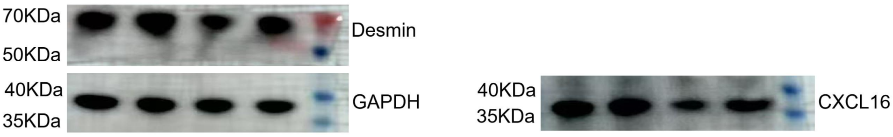

labeling：the grouping of gels/blots cropped from different parts of the same gel

2.Full-length gel/blot for Figure 4 and 7

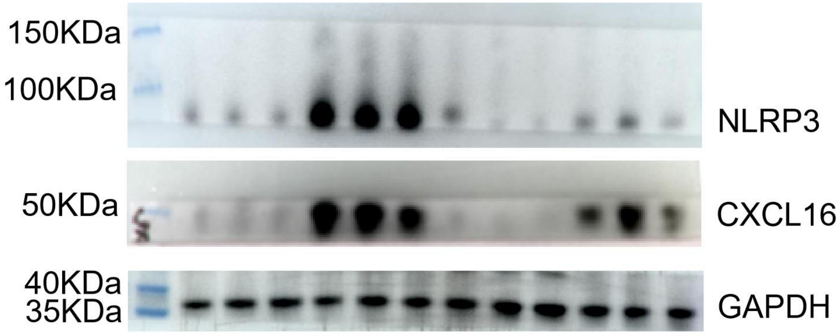

labeling：the grouping of gels/blots cropped from different parts of the same gel

3.Full-length gel/blot for Figure 6

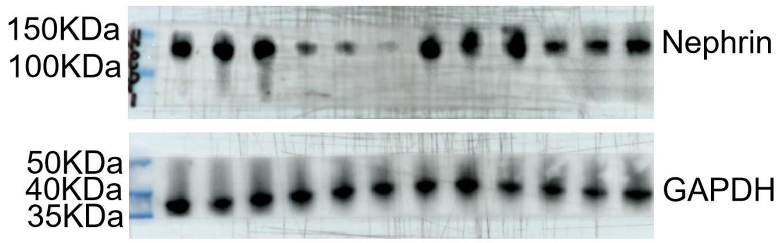

labeling： the grouping of gels/blots cropped from different parts of the same gel

4. The ethical approval for human research

山东大学临床医学院伦理委员会

审　批　件

编号：SDULCLL2021-1-32

项目名称：P2X7R-NLRP3-IL-1β通路激活CXCL16在肾小球硬化中作用机制的研究

项目负责人：孙书珍　　职称：教授　　联系电话：13791093267

负责研究单位：山东大学临床医学院

合作研究单位：日照市人民医院

研究起止时间：2023.01.01-2025.12.31

拟申报项目类别：山东省自然科学基金面上项目

审查意见：

经本委员会审查，研究者的资格、经验符合试验要求；研究方案符合《赫尔辛基宣言》以及《涉及人的生物医学研究伦理审查办法》等国际国内法律和有关伦理规范的要求；知情同意方法适当；受试者可能遭受的风险与研究预期的受益相比适当。

同意开展该项目的研究，但在研究过程中应接受本委员会的监督，研究者在研究结束后，应当向本委员会递交最终报告，包含对于研究发现及研究结论的总结。

山东大学临床医学院伦理委员会

主任委员：

2021年11月18日

地址：济南市文化西路44号山东大学趵突泉校区

联系电话：（+86 531）88382709　　E-mail：chengzhu@sdu.edu.cn
