# Supplementary material for: Inhibiting the P2X7R-NLRP3 inflammasome pathway regulates CXCL16 to alleviate podocyte injury in mice with adriamycin nephropathy
Source: Sci Rep. 2026 Apr 5;16:16361. doi: 10.1038/s41598-026-47345-5 (PMC13212586; doi:10.1038/s41598-026-47345-5)
Supplement: Supplementary file 2 — Supplementary Information 2. [file 41598_2026_47345_MOESM2_ESM.pdf]

1.Full-length gel/blot for Figure 2

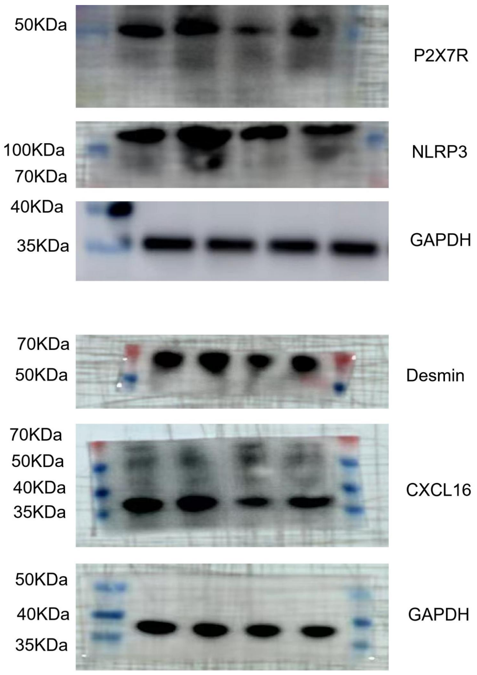

2.Full-length gel/blot for Figure 4 and 7

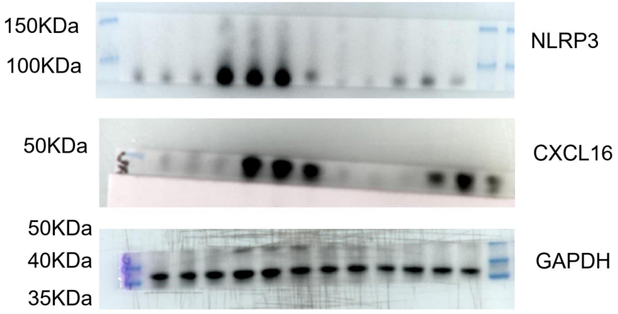

3. Full-length gel/blot for Figure 6

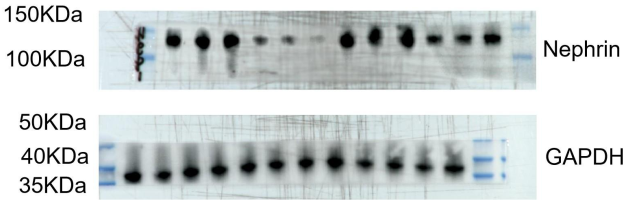

4. The ethical approval for animal experiments

山东大学临床医学院伦理委员会
动物实验伦理审查审批件

编号： SDULCLL2021-2-32

项目名称：P2X7R-NLRP3-IL-1β 通路激活 CXCL16 在肾小球硬化中作用机制的研究

项目来源：山东省自然基金面上项目

项目负责人：孙书珍　　　　　　　联系电话：13791093267

负责研究单位：　山东大学临床医学院

合作研究单位：　日照市人民医院

研究起止时间：　2023.01.01-2025.12.31

审查意见：

经本委员会审查：该项目对实验动物的使用符合动物实验伦理的"3R"原则，研究单位具备相关资质，实验相关人员具备从事动物实验的相关资格。实验所用动物品种品系合适。实验设计中未发现有违反中华人民共和国国家科学技术委员会制定的《实验动物管理条例》、中华人民共和国科学技术部发布的《关于善待实验动物的指导性意见》之处。实验设计符合动物实验相关伦理原则的要求。

本委员会同意项目负责人开展该项目的研究，但在实验过程中应注意维护实验动物的有关福利，并接受本委员会的监督。

山东大学临床医学院伦理委员会

山东大学临床医学院伦理委员会

主任委员：JTK

2021 年 11 月 18 日

地址：济南市文化西路 44 号山东大学趵突泉校区

联系电话：（+86 531）88382709　　E-mail：chengzhu@sdu.edu.cn
